# Supplementary material for: SHIV remission in macaques with early treatment initiation and ultra long-lasting antiviral activity
Source: Nat Commun. 2024 Dec 4;15:10550. doi: 10.1038/s41467-024-54783-0 (PMC11618496; doi:10.1038/s41467-024-54783-0)
Supplement: Supplementary file 1 — Supplementary Information [file 41467_2024_54783_MOESM1_ESM.pdf]

Supplementary Materials

SHIV remission in macaques with early treatment initiation and ultra long-lasting antiviral activity

Michele B. Daly[1], Chuong Dinh[1,] Angela Holder[1], Donna Rudolph[1], Susan Ruone[1], Alison Swaims-Kohlmeier[1,6], George Khalil[2,7], Sunita Sharma[1], James Mitchell[1], Jillian Condrey[3], Daniel Kim[1], Yi Pan[2], Kelly Curtis[1], Peter Williams[4,8], William Spreen[5], Walid Heneine[1], J. Gerardo García-Lerma[1]*

[1]Laboratory Branch, Division of HIV Prevention, National Center for HIV, Viral Hepatitis, STD, and TB Prevention, Centers for Disease Control and Prevention; Atlanta, GA 30329, USA
[2]Quantitative Sciences and Data Management Branch, Division of HIV Prevention, National Center for HIV, Viral Hepatitis, STD, and TB Prevention, Centers for Disease Control and Prevention; Atlanta, GA 30329, USA
[3] Comparative Medicine Branch, Division of Scientific Resources, National Center for Emerging and Zoonotic Infectious Diseases, Centers for Disease Control and Prevention; Atlanta, GA 30329, USA
[4]Janssen Research & Development, Beerse, Belgium
[5] ViiV Healthcare, Research Triangle Park, NC 27709, USA.
[6]Current affiliation: Department of Microbiology & Immunology, Emory University School of Medicine; Atlanta, GA 30322, US
[7]Current affiliation: Office of Informatics and Data Management, Centers for Disease Control and Prevention; Atlanta, GA 30329, USA
[8]former Janssen employee (retired)

*Corresponding author. Email: jng5@cdc.gov

Supplementary Figures and Tables

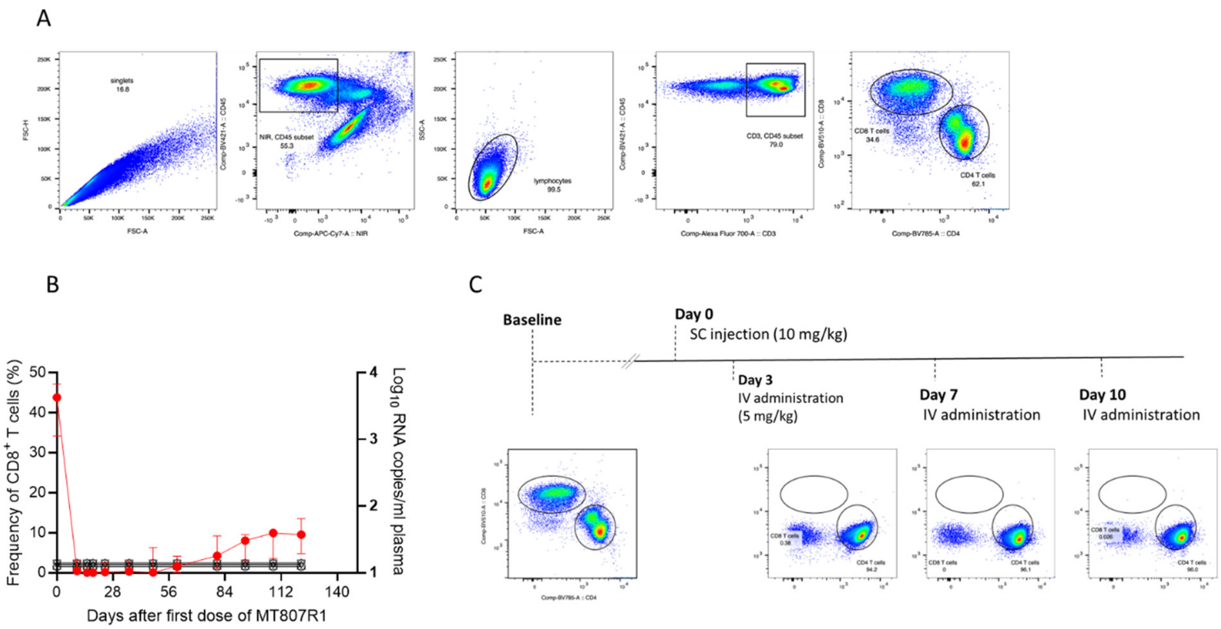

Supplementary Fig. 1. Antibody-mediated CD8+ T cell depletion after treatment cessation is not associated with virus rebound. A: Gating strategy. Viable singlet CD45+ cells were discriminated for lymphocytes using forward and side scatter characteristics. **B:** Macaques (n=4) received a subcutaneous dose (10 mg/kg) of anti-CD8 monoclonal antibody MT807R1 at 29 months post infection (or 17 months after treatment cessation) followed by three subsequent intravenous doses (5 mg/kg) 3, 7, and 10 days later. Figure plots plasma SHIV RNA levels for each individual animal (dark lines, right axes) and median (range) CD8+ T cells (red line, left axis) seen in the 4 macaques overtime. **C:** Representative FACS results from a macaque treated with MT807R1. Source data are provided as a Source Data file.

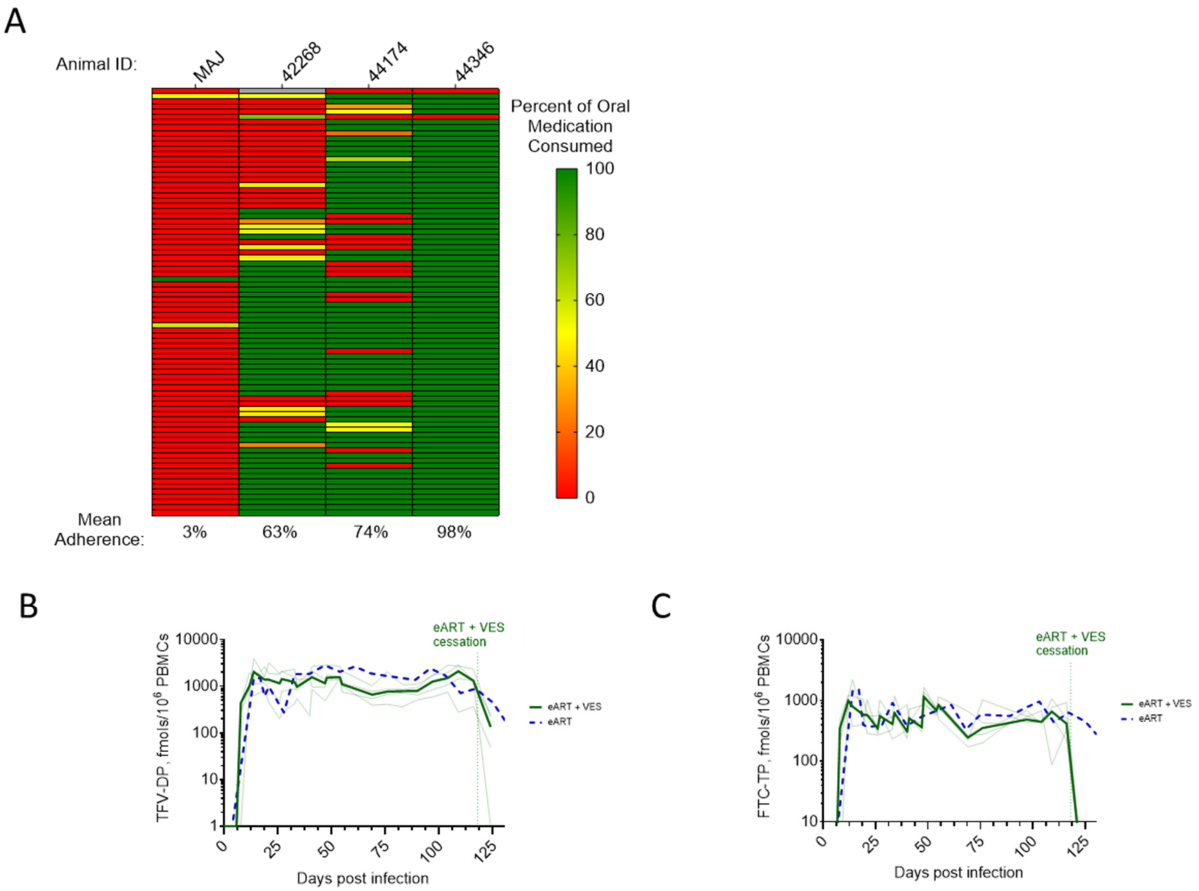

Supplementary Fig. 2. Adherence to oral FTC/TAF in the eART + VES animals and intracellular drug concentrations in PBMCs. A) adherence diaries to oral FTC/TAF for each individual animal of the eART + VES group. On the days that oral medication was no consumed, FTC/TAF was delivered subcutaneously to provide full drug coverage. **B)** TFV-DP concentrations seen in each individual macaque treated with eART + VES (light green). **C)** FTC-TP concentrations seen in each individual macaque treated with eART + VES (light green). Solid lines in panels B and C denote the median TFV-DP and FTC-TP concentrations seen in the four eART + VES-treated macaques (dark green) and the four eART-treated (dark blue) macaques. Source data are provided as a Source Data file.

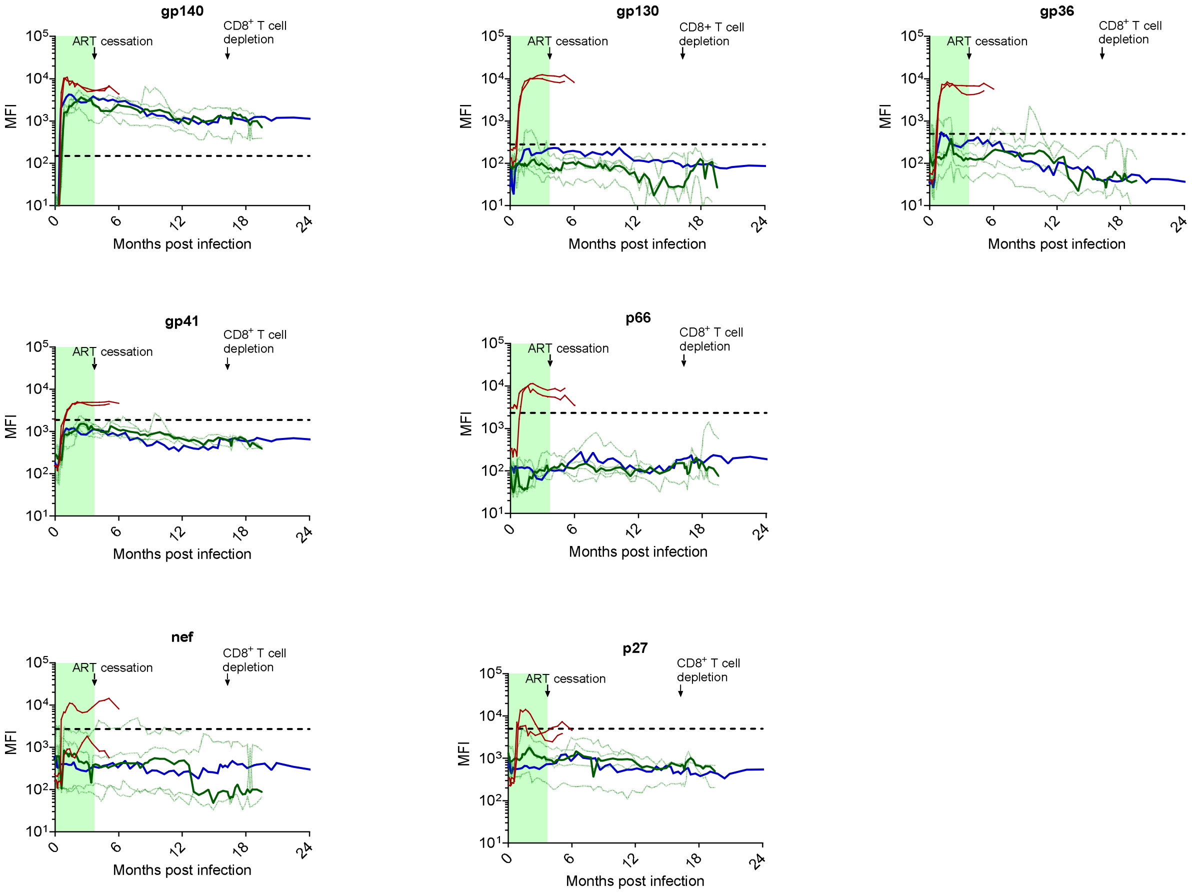

Supplementary Figure 3. Longitudinal analysis of SHIV antibody levels in animals treated with eART + VES. SHIV plasma IgG levels against gp140, gp130, gp41, p66, nef and p27 were measured longitudinally using a Bio-Plex assay. Light green lines denote the median fluorescent intensity (MFI) for each individual macaque treated with eART + VES; dark green line indicates the median value for the 4 eART + VES animals. Two untreated controls are shown in red. Median MFI values from the eART alone group are also shown for comparison (blue line). The shaded green area represents the period of treatment with eART (months 1-4) + VES (months 1-3). The horizontal dotted line denotes cutoff values for each analyte. Source data are provided as a Source Data file.

Supplementary Table 1. Tissue concentration of CAB and RPV measured 3 weeks after dosing.

Tissue	CAB (ng/g)	RPV (ng/g)
Lymphoid tissue		
Inguinal lymph nodes	975	692
Axillary lymph nodes	412	773
Mesenteric lymph nodes	23,669	28,800
Iliac lymph nodes	1,279	736
Cervical lymph nodes	583	1,772
Gastrointestinal tract		
Duodenum	569	666
Jejunum	643	697
Ileum	695	518
Cecum	1,367	670
Rectum	773	447
Brain tissues		
Cerebellum	58	259
Basal ganglia	26	340
Spinal cord	288	437

Supplementary Table 2. Antibodies used for flow cytometry.

Antibody	**Conjugate**	**Clone**	**Vendor**
**CD3**	Alexa Fluor® 700	SP34-2	BD Biosciences
**CD4**	Brilliant Violet 785™	OKT4	Biolegend®
**CD8**	Brilliant Violet 510™	RPA-T8	BD Biosciences
**CD45**	BD Horizon™ BV450	D058-1283	BD Biosciences
